# Cognitive deficits following exposure to pneumococcal meningitis: an event-related potential study

**DOI:** 10.1186/1471-2334-12-79

**Published:** 2012-03-31

**Authors:** Michael Kihara, Michelle de Haan, Eugene O Were, Harrun H Garrashi, Brian GR Neville, Charles RJC Newton

**Affiliations:** 1 The Centre for Geographical Medicine Research (Coast), Kenya Medical Research Institute, Kilifi, Kenya; 2 Developmental Cognitive Neuroscience Unit, University College London Institute of Child Health, London, UK; 3 Department of Paediatrics, University of Nairobi, Kenyatta National Hospital, Nairobi, Kenya; 4 Neurosciences Unit, University College London Institute of Child Health, The Wolfson Centre, London, UK; 5 London School of Hygiene and Tropical Medicine (LSHTM), London, UK

## Abstract

**Background:**

Pneumococcal meningitis (PM) is a severe and life-threatening disease that is associated with cognitive impairment including learning difficulties, cognitive slowness, short-term memory deficits and poor academic performance. There are limited data on cognitive outcomes following exposure to PM from Africa mainly due to lack of culturally appropriate tools. We report cognitive processes of exposed children as measured by auditory and visual event-related potentials.

**Methods:**

Sixty-five children (32 male, mean 8.4 years, SD 3.0 years) aged between 4-15 years with a history of PM and an age-matched control group of 93 children (46 male; mean 8.4 years, SD 2.7 years) were recruited from a well-demarcated study area in Kilifi. In the present study, both baseline to peak and peak-to-peak amplitude differences are reported.

**Results:**

Children with a history of pneumococcal meningitis had significantly longer auditory P1 and P3a latencies and smaller P1 amplitudes compared to unexposed children. In the visual paradigm, children with PM seemingly lacked a novelty P3a component around 350 ms where control children had a maximum, and showed a lack of stimulus differentiation at Nc. Further, children with exposure to PM had smaller peak to peak amplitude (N2-P1) compared to unexposed children.

**Conclusion:**

The results suggest that children with a history of PM process novelty differently than do unexposed children, with slower latencies and reduced or absent components. This pattern suggests poorer auditory attention and/or cognitive slowness and poorer visual attention orienting, possibly due to disruption in the functions of the lateral prefrontal and superior temporal cortices. ERPs may be useful for assessment of the development of perceptual-cognitive functions in post brain-injury in African children by providing an alternate way of assessing cognitive development in patient groups for whom more typical standardized neuropsychological assessments are unavailable.

## Background

Pneumococcal meningitis (PM) is an infection of the membranes covering the brain and spinal cord caused by *Streptococcus pneumoniae *[1]. The case fatality rate for PM in resource-constrained countries varies from 12-50% [2,3], which is much higher than in developed countries, and much more than other forms of bacterial meningitis [2]. Cognitive dysfunction following the infection is reported [4-9] including learning difficulties, cognitive slowness, short-term memory deficits and poor academic performance [10-13].

Methodological limitations contribute to the paucity of data on cognitive outcomes of children following PM in Africa. Neuropsychological assessments are arguably the most important tools [14], but in Africa, lack of standardization, low literacy levels, language and cultural barriers [15,16] limit successful testing [17].

Event related potentials (ERPs) are electroencephalographic changes that are time-locked to sensory and cognitive events and represent the neurophysiologic processing of these events [18]. ERPs reflect the activation of neural structures in the sensory cortex, cortical association areas and higher order cognitive areas [19]. They measure basic sensory processing abilities related to cognition and have been used successfully in studying the effects of malaria in Kenya [20]. In children, the P1, the N2 and the P3a are the typical components observed in an auditory novelty oddball [21-23]. In a visual paradigm, the common components in children are the N1 [24], the P2 [25], the P3a [26] and the Nc [27,28] at midline electrodes and, if face stimuli are used, a face-sensitive N170 at occipito-temporal sites [29]. Previous studies suggest that these ERP components may be clinically useful as an index of cognitive function [30]. We therefore recorded novelty processing in the auditory and visual modality and compared children with and without prior history of PM.

## Methods

### Subjects

Children with a history of PM were selected from the database of admissions to the paediatric ward at Kilifi District Hospital (KDH). PM was defined as cerebrospinal fluid culture (CSF), gram stain or latex antigen test positive for *Streptococcus pneumonia*. The children were resident in the Kilifi Demographic Surveillance System (DSS) to ensure that they could be identified in the community. At the time of assessment written consent was obtained from the parents/guardian. Exclusion criteria were presence of pre-existing congenital neurological conditions i.e. spina bifida, history of severe birth asphyxia or jaundice requiring medical intervention (phototherapy or exchange transfusion). Community controls were matched for sex and age (within 3 months), were residents in the Kilifi DSS for more than one year and had informed consent from parent/guardians. Those with a history of severe birth asphyxia, pre-existing neurological conditions and a history of neonatal jaundice were excluded. The study was approved by the National Ethics Committee.

**Figure 1:**
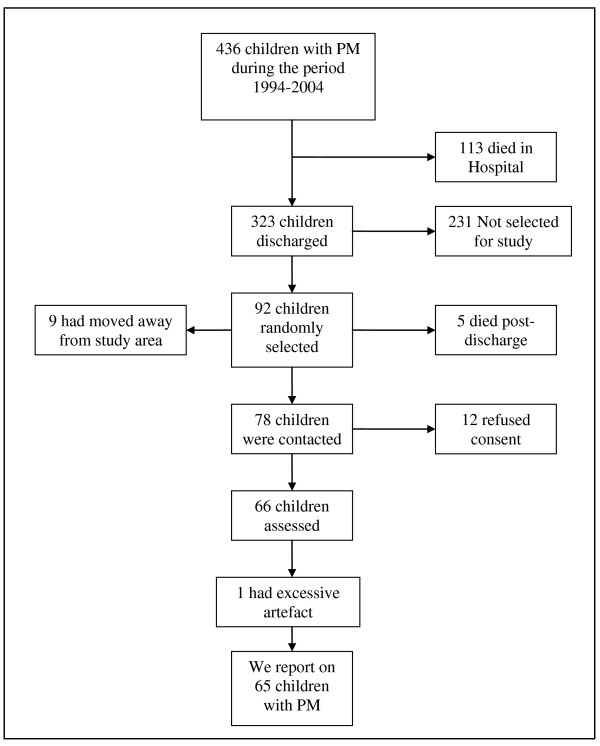
**Selection process of children with a history of pneumococcal meningitis**.

There were 436 children admitted with PM at KDH during the period 1994-2004, of whom 113 (26%) died in hospital (Figure 1). A sample size of 80 children was required to detect a difference between cases and controls with 90% power and 95% confidence interval [31]. To account for potential 15% failure-rate in recruitment, we generated a random list of 92 children who had been admitted to Kilifi District Hospital with PM from 1994 to 2004. Of those followed up 5 (5.4%) had died after discharge, 9 (9.2%) had migrated out of the study area and 12 (13.2%) refused consent. Of the 66 who consented to the study, 1 child's ERP data was not analyzed due to excessive artefact. Sixty-five children (32 male; 33 female) aged between 4-15 years old (mean 8.4 yr, SD 3.1 yr) with a history of PM were included. A control group of 93 children (46 male; 47 female) aged between 4-15 years old (mean 8.4 yr, SD 2.6 yr) years was recruited from a well-demarcated study area in Kilifi from the DSS maintained in the KDH.

### Visual and auditory screening

The visual and hearing screening was performed using the Sonksen-Silver [32] and Kamplex KS16 screening audiometer (P.C. Werth, UK) respectively. Auditory screening took place in a sound attenuated room to minimize environmental noise. Air conduction at 500 Hz, 1000 Hz, 2000 Hz and 4000 Hz in each ear was carried out according to the recommendations of British Society of Audiology (BSA, 1988). The hearing impairment was classified according to the lowest tone the ear could hear: mild impairment 26-40 dB, moderate impairment 41-60 dB, severe impairment 61-80 dB and profound impairment > 81 dB.

Children with severe (unable to hear 61 - 80 dB on better ear) to profound (over 81 dB) hearing impairment were excluded from the auditory paradigm analyses, resulting in seven children (7.7%) being excluded from the PM group and none from the control group.

### ERP Recording

Children who were 5 years-old or younger had electrodes individually positioned at the midline brain locations (i.e. Fz, FCz, Cz and Pz) as well as A1, A2, HEOG, VEOG and Fpz using the international 10-20 system [33]. In the older children, a greater number of electrodes were used for recording including temporal sites (i.e. F7, F8, T3, T4, T5, T6, P3, and P4), frontal (i.e. Fp1 and Fp2) and mastoids (i.e. A1 and A2). All electrodes were referenced to Cz with Fpz as the ground electrode but re-referenced offline to linked mastoids.

The auditory paradigm was composed of three types of sounds: frequent and infrequent pure sinusoidal tones, and novel sounds. These tones and novel sounds were presented through two speakers placed in front of the children. Ten percent of the stimuli were infrequent tones (2000 Hz, 200 ms long, 5 ms rise and fall time, 70 dB Sound Pressure Level, SPL), 10% were composed of novel noises e.g. dog bark, bell ring, etc. whereas the remainder were frequent tones of (1500 Hz, 200 ms long, 5 ms rise and fall time, 70 dB SPL). The duration of the tones/noises was 200 milliseconds (ms) with a stimulus onset asynchrony of 700 ms. Two-blocks of 700 stimuli each were presented (560 frequent, 70 infrequent and 70 novels). Novel sounds were digitally adjusted in intensity so that they did not exceed 70 dB SPL as determined using a Bruel and Kjaer sound pressure meter. The frequent stimuli immediately prior to each infrequent stimulus were selected for averaging to provide similar signal-to-noise ratios.

The visual paradigm consisted of three kinds of images: an infrequently presented face and a frequently presented face (both were photographs of African women), and infrequently presented trial-unique, non-face abstract patterns (i.e. photographs of Kandinsky's paintings). Stimuli were of equal size and presented at a visual angle of 16.78 × 14.25 degrees. Two blocks of 100 trials were presented in a random order, with 60% of the trials showing the frequent face, 20% infrequent face, and 20% non-face abstract picture stimuli (trial unique). Participants were asked to look at a cross at the centre of the screen to minimize eye-movement artefacts. The duration of the image presentation was 500 ms with an inter-stimulus interval of 3000 ms. In total, the tasks took between 20-30 minutes to complete.

Offline analyses of auditory data were collected using a 700 ms recording epoch with a 200 ms pre-stimulus baseline while the visual data had a 1500 ms recording epoch and a 200 ms baseline. EEG data were low-pass filtered offline at 20 Hz artefact-rejected (for blinks and muscle activity), baseline corrected, and waveforms were divided into epochs centred on stimulus presentation. An ocular artefact reduction algorithm on the Scan 4.3 software (Neuroscan^® ^Labs) was used to remove artefacts due to blinking. Any trials with amplitude deflections exceeding +/-100 μV were rejected. A minimum of 20 trials for each stimulus was required for inclusion of an individual average ERP waveform.

In the auditory paradigm, the P1 component was defined as the highest peak between 60 and 130 ms post stimulus presentation and the N2 component was defined as the most negative point between 120 and 220 ms. The P3a component was defined as the most positive point occurring between 250 and 450 ms.

In the visual paradigm, the N170 was defined as the most negative peak between 170-250 ms and was measured at occipito-temporal sites T5 and T6. The P3a was the highest peak between 270 and 450 ms and Nc was the average amplitude between 300 and 850 ms.

All analysis was conducted using Predictive Analysis Software for Windows, version 18 (SPSS Inc^®^, Chicago, USA). Within-subject factors included site (X4: Fz, FCz, Cz and Pz) and stimuli (X3: frequent, infrequent and novel). The between-subject factors were age (X4: 4-5, 6-7, 8-9, 10-15) and sex (X2: male or female). The Greenhouse-Geisser correction is reported where applicable. We used the Tukey-Kramer test in the *post-hoc *analyses to correct for unequal sample sizes. Level of significance was set at p < 0.05.

## Results

### Auditory ERPs

**Figure 2:**
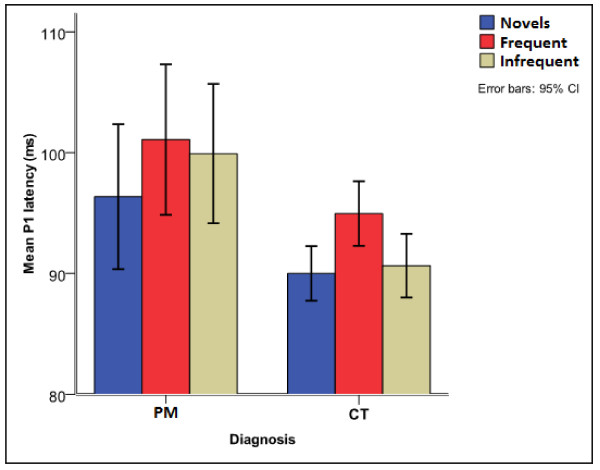
**Auditory P1 latency for children with exposure to PM and controls**.

**Figure 3:**
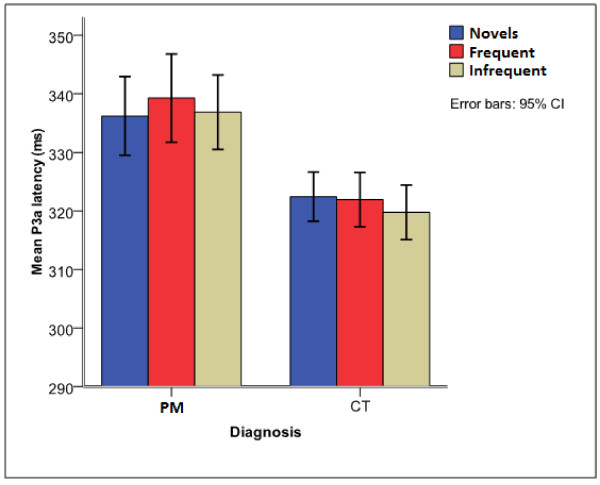
**Comparison of mean P3a latency between children with exposure to PM and unexposed children**.

Children with exposure to PM had consistently longer auditory ERP latencies (Figures 2 and 3), but comparable amplitudes with unexposed children except for the amplitude of the P1 component in younger children with PM. A more detailed comparison of the diagnostic groups on the various components is presented.

#### Auditory P1

**Figure 4:**
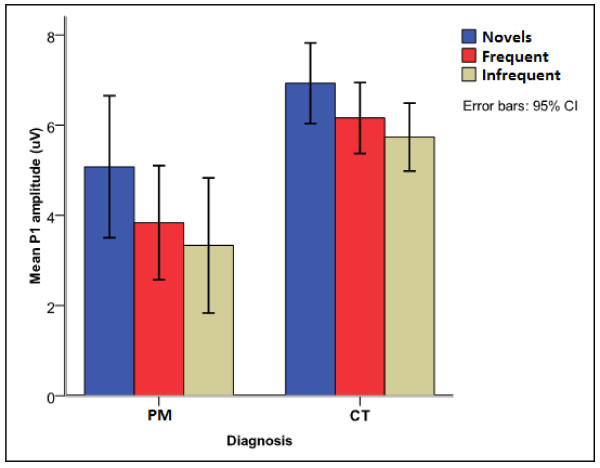
**Auditory P1 amplitude for children with exposure to PM and controls**.

Exposure to PM significantly affected the P1 latency [F (1, 176) = 10.72, p < 0.015]. This occurred due to longer mean P1 latency (99 ± 22.5 ms vs 90 ± 11.0 ms) in children with exposure to PM compared to unexposed children [t (190) = -2.991, p = 0.003] (Figure 2). The amplitude for the P1 component had a main effect of diagnosis [F (1, 176) = 5.530, p = 0.020] and an interaction effect of electrode by diagnosis by age [F (12, 408) = 2.607, p = 0.014]. The main effect occurred due to significantly smaller P1 amplitude in children exposed to PM compared to unexposed children (Figure 4). The interaction occurred due to significantly smaller amplitudes for the PM children at the Pz site for 4-5 and 6-7 year-olds [3.3 ± 2.7 vs 1.24 ± 2.2 μV, p = 0.018 and 4.5 ± 3.2 vs 1.8 ± 4.7 μV, p = 0.047 respectively].

#### Auditory N2

**Figure 5:**
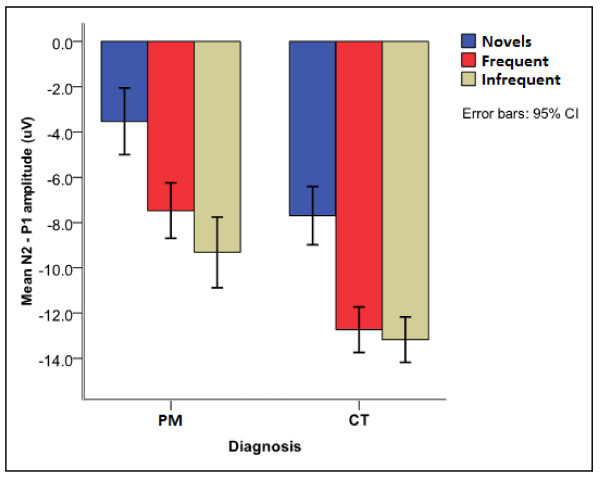
**Mean auditory N2-P1 amplitude for children with exposure to PM and controls**.

The N2 latency had significant interaction effect of stimulus by diagnosis by age [F (8, 272) = 2.560, p = 0.012]. The interaction effect of stimulus by diagnosis by age occurred due to a significantly faster N2 latency for novel stimuli for unexposed children at age 10-15 years compared to younger ages [4-5 y > 10-15 y, p = 0.021; 6-7 y > 10-15 y, p = 0.019; 8-9 y > 10-15 y, p = 0.024] but not for children exposed to PM [p = 0.248, p = 0.361 and p = 0.803 respectively]. The N2 amplitude from baseline to peak did not differentiate between children with exposure to PM and unexposed children. However, the peak to peak amplitude (N2-P1) had main effects of age [F(4, 183) = 14.011, p < 0.001] and diagnosis [F(1, 183) = 10.445, p = 0.001]. The main effect of age occurred due to decreasing amplitude differences with age. The main effect of diagnosis was due to significantly smaller amplitude differences for children exposed to PM compared to unexposed children (Figure 5).

#### Auditory P3a

The P3a latency had a significant main effect of diagnosis [F (1, 136) = 6.112, p = 0.015]. This occurred due to longer P3a latency (333 ± 29.7 ms vs 321 ± 15.1 ms) in children with exposure to PM compared to unexposed children [t (190) = -2.955, p = 0.004] (Figure 3). The P3a amplitude both from baseline to peak and from peak to peak (P3a - N2) did not distinguish between the two diagnostic groups. There was no significant main effect of stimulus.

### Visual ERP

**Figure 6:**
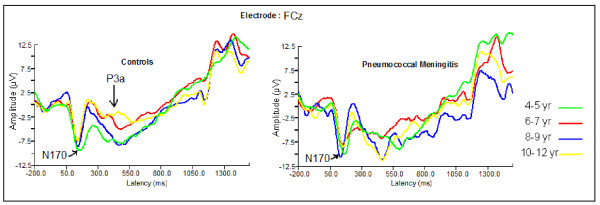
**Comparison of visual novelty processing between children exposed to pneumococcal meningitis and unexposed children by age**.

Children with exposure to PM had a slightly different pattern of visual waveforms compared to unexposed children (Figure 6). The development of the ERPs by age that is evident in the waveforms of unexposed children was absent in children exposed to PM. The P3a component that occurs around 350 ms in controls was all but absent in the PM groups except in the older children (Figure 6), and the Nc component showed less differentiation among the visual stimuli in the unexposed compared to exposed group.

**Figure 7:**
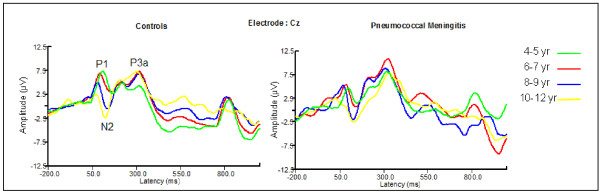
**Comparison of auditory novelty processing between children exposed to pneumococcal meningitis and unexposed children by age**.

#### Visual N170

The latency and amplitude of the N170 component did not distinguish between PM and unexposed children.

#### Visual P3a

In the grand averaged waveforms of the children exposed to PM, there lacked a visual P3a component around 350-450 ms where it existed in the control group (Figure 5). In absence of the group averaged component, we did not study this component any further.

#### Visual Nc

The Nc revealed interaction effects of stimulus by diagnosis [F (2, 274) = 3.952, p = 0.026] and stimulus by electrode by diagnosis [F (6, 822) = 4.460, p = 0.004]. The interaction of stimulus by diagnosis occurred due to larger amplitude associated with infrequent stimuli compared to frequent stimuli in unexposed children [2.7 ± 5.4 vs 0.8 ± 3.6 μV, p = 0.001] but not in children with a history of PM [p = 0.386]. The interaction of stimulus by electrode by diagnosis was due to larger amplitudes associated with infrequent compared to frequent stimuli at Fz, Cz and Pz [p = 0.026, p < 0.001 and p < 0.001] for unexposed children but not for children with a history of PM [p = 0.479, p = 0.884 and p = 0.136 respectively].

## Discussion

The present study used auditory and visual event related potentials to novelty in children exposed pneumococcal meningitis, to investigate whether the disease disrupted the neural processes underlying visual and auditory attentional orienting, and its possible effects on brain processing as a marker of cognitive function [34].

Children exposed to PM had longer auditory P1 latencies than unexposed children. The P1 component has been thought to be an objective measure of cortical auditory function [35,36] or preferential attention [37] in children. In general, there are age-related decreases in P1 latency with increasing age, which is shown in children studies [38-40]. Longer P1 latencies in children with exposure to PM suggest slower or impaired development of their auditory functions. The P1 component is thought to be generated by the superior temporal gyrus [41]. The superior temporal gyrus contains several important structures of the brain, including those that deal with the sensation of sound and Wernicke's area (processing of speech). Previous studies show that children with a history of bacterial meningitis were at risk for language difficulties (post illness) [5,42]. The results may suggest that PM may result in a delayed processing perhaps due to subtle hearing loss resulting in longer P1 latencies in children with exposure to PM [36].

The auditory N2 latency is influenced by attention and task difficulty [43,44]. It is thought to reflect focused attention to stimulus features [45] and is likely to index stimulus classification. Unexposed children showed decrease in the latency of the N2 component with age, but those exposed to PM had longer N2 latencies with age. The decrease of the N2 latency with age in unexposed children has been shown in previous studies [46-48]. This decrease is thought to represent maturational changes probably due to decreased synaptic density and an advance of intra-cortical myelination [38]. The N2 component is said to result from a deviation in form or context of a prevailing stimuli [49]. In the present study, novel sounds (environmental noises) provided a deviation from the standard tones (1000 Hz, 70 dB SPL) and target tones (2000 Hz, 70 dB SPL) which acted as the prevailing stimuli. The auditory N2 is thought to originate bilaterally in the auditory cortex of the superior temporal lobes with frontal predominance [50]. It reflects attention orienting and its decrease with age may reflect inhibitory attention control [51].

The P1-N2 peak to peak amplitudes to novel stimuli showed significant differences between children with exposure to PM and unexposed community children. This aimed at determining whether the amplitude of the N2 component was influenced by the diagnosis of the children independent of the P1 component. The smaller brain responses in children with exposure to PM suggests deficits in cognitive processes necessary to discriminate novel stimuli [51,52].

The P3a component reflects an involuntary attention switch from the actual focus of voluntary attention to the eliciting sound [53], and is generated in the frontal lobes [54-56]. The P3a has also been interpreted as a neural correlate of the orienting response [57]. Our study results showed that children with exposure to pneumococcal meningitis had longer auditory P3a latencies compared to unexposed children. Studies have reported a decrease in P3a latencies with age [46,58-61] between 5 and 16 years but some have found no difference between 5 and12 years [62]. The results of the present study show that children with exposure to PM had much longer P3a latencies which can be attributed to impaired orientation to novelty, cognitive slowness or inflexible set-shifting. The P3a is recorded at the anterior locations and we speculate that PM does impair neural pathways in the frontal lobes, especially those dealing with executive functions [63].

The present study revealed absence of the visual P3a component in children exposed to PM. Some previous studies have found marked reduction and even absence of this component in patients with alcoholism [44,64] and epilepsy patients [65]. The P3a component is thought to reflect automatic orienting and so its absence in children with exposure to PM is particularly interesting. Future studies should seek to study this component using an active paradigm.

Generally, amplitudes are related to the strength of the neural association while the latencies represent the time to respond to the stimuli [66]. The results of the latency differences suggest slowness or distractibility in children exposed to PM but not absence of response. Bacterial meningitis is thought to disrupt on-going brain maturation in developing children [5]. Frontal lobe development and myelination are critical and interruption of this process results in slowed information processing and poor development of executive functioning [67,68].

Previous research has reported potentially serious cognitive consequences in children with a history of pneumococcal meningitis [6,12,42]. These children have lower IQ's and performed poorer on neuropsychological tests than their peers many years post infection. Damage to frontal lobes leads to diminished attention through disruption of neural process [69,70]. The results of the present study thus suggest that pneumococcal meningitis impairs neural development in the frontal lobes, causing negative effects on children's cognitive development in general and affecting their academic outcomes.

### Limitation of the study

The results of the auditory paradigm of study should be interpreted with caution since bacterial meningitis is known to cause subtle to profound hearing loss and the children were not fitted with hearing aids. Children with severe to profound hearing loss were excluded from the ERP paradigms to minimize obvious biases arising from sensory impairments. However, it is possible that subtle hearing loss and cortical blindness could have accounted for the differences in children with pneumococcal meningitis. The auditory N2 and visual P2 latencies are indices of processing speed and attention allocation and could have been affected by subtle impairments.

Also, a lack of neuropsychological testing in this study meant that we could not compare the differences in function between children exposed to PM and those who were unexposed.

Studies in Africa have shown a high HIV prevalence in children with meningitis [71]. However, the children in the present study did not have their HIV status investigated and their sero-status may have influenced the study results.

## Conclusion

The results of the passive ERP paradigm show that there is a difference between children exposed to pneumococcal meningitis and unexposed children. These exposed children generally have a slower brain processing as depicted by their longer ERP latencies of the various components. The results suggest impaired development of frontal cortex and/or its connecting pathways, possibly due to disruption of brain development by meningitis infection. This methodology may be useful for assessment of the development of perceptual-cognitive functions in African children providing an alternate way of assessing perceptual-cognitive development in patient groups for whom more typical standardized neuropsychological assessments are unavailable.

## Competing interests

The authors declare that they have no competing interests.

## Authors' contributions

MK, EOW, BGRN and CRJCN were responsible for the conception of the study, its design, data collection and critical review of the manuscript. HH and MK were involved in the recording and analysis of the event-related potentials. MdH, CRJCN and MK were involved in the evaluation and interpretation of results. MK wrote the initial draft of the manuscript and all other authors contributed substantially to the content. All authors have read and approved the final manuscript.

## Pre-publication history

The pre-publication history for this paper can be accessed here:

http://www.biomedcentral.com/1471-2334/12/79/prepub
